# Preference-Performance Dissociation in Golf Putting

**DOI:** 10.3389/fpsyg.2020.00102

**Published:** 2020-01-29

**Authors:** Gal Ziv, Ronnie Lidor, Liav Elbaz, Matar Lavie

**Affiliations:** Motor Behavior Laboratory, The Academic College at Wingate, Wingate Institute, Netanya, Israel

**Keywords:** skill acquisition, golf putting, autonomy, preference-performance dissociation, motor learning

## Abstract

The purpose of the current study was to examine the effectiveness of providing autonomy to learners and the phenomenon of preference-performance dissociation on a closed, self-paced motor task – putting in golf, when using different placements of a visual aid (a large circle) around a golf hole. Seventy-six participants were assigned to four experimental groups: (a) a visual aid placed behind the hole (V-behind group), (b) a visual aid placed in front of the hole (V-in-front group), (c) a visual aid placed around the hole (V-around group), and (d) a visual aid placed according to the participant’s preference (V-pref group). Participants performed five pre-trials, 50 training putts from a distance of 2 m, a retention task (12 putts) from a distance of 2 m, and a transfer task (12 putts) from a distance of 2.5 m. The retention putts and transfer putts were performed 48 h after the training putts. The participants’ subjective assessment of the helpfulness of the circle was also measured. It was found that in the retention task, putting consistency was lower in the V-in-front group compared to the V-around and V-pref groups. However, the subjective assessment of the helpfulness of the circle was higher in the V-in-front group. In addition, the low consistency of the V-in-front group was alleviated in the participants in the V-pref group who chose to place the circle in front of the hole. In contrast, the subjective assessment of the helpfulness of the circle was low in the V-in-front group. These findings suggest that while providing autonomy – that is, when the participant is able to choose for him/herself – can improve motor learning, there may be a dissociation between an individual’s subjective assessment and the actual helpfulness of a visual aid. This dissociation may be termed *preference-performance dissociation*, and coaches and instructors who teach closed, self-paced motor skills should be aware of the fact that when providing learners with the autonomy to choose a practice aid in order to improve their skills, some may not choose the aid that is effective for them.

## Introduction

Closed, self-paced motor tasks are those taking place in a relatively stable and predictable environment, where there is adequate time to prepare for their execution (Lidor, 2007; Schmidt et al., 2018). Among these tasks are shooting in archery, free-throw shooting in basketball, and golf putting. Beginning learners who attempt to acquire closed, self-paced motor tasks can benefit from the use of visual aids. For example, beginner golfers can find relevant instructional information on how to use various visual aids in order to (a) prepare themselves for the putting act, (b) aim the ball at the hole, and (c) control the speed and direction of the ball (see, e.g., Farnsworth, 1997; Pelz, 2005, 2012).

One of the visual aids that has been found to improve performance in closed, self-paced motor tasks is the use of a large circle around the target. The presence of a large circle (as opposed to a small circle) around a target has been shown to enhance aiming or putting accuracy (Palmer et al., 2016; Ziv et al., 2019). This phenomenon can be explained by the concept of *enhanced expectancies of success*, which suggests that when one has enhanced expectancies of success, one actually performs better. A number of possible underlying mechanisms for the benefits of enhanced expectancies are: (a) increased positive affect and self-efficacy; (b) improved preparation for task performance; and (c) improved outcome expectations and prediction of external rewards (Wulf and Lewthwaite, 2016). The abovementioned mechanisms suggest that expectations for good performance may “prepare the mover for successful movement through diverse effects at cognitive, motivational, neurophysiological, and neuromuscular levels – ensuring that goals are effectively coupled with desired actions” (Wulf and Lewthwaite, 2016, p. 1390).

Two observations can be made based on the placement of a large circle around the target in the studies mentioned in the previous paragraph (i.e., Palmer et al., 2016; Wulf and Lewthwaite, 2016; Ziv et al., 2019): (a) the circle was placed symmetrically around the target or the golf hole, and (b) the experimenter was the one to determine the placement of the large circle around the target. Indeed, placing the visual aid symmetrically around the target assisted the participants in improving their performance. However, placing the visual aid in a non-symmetrical manner may have had a differential effect on performance. Beginner golfers, like other individuals who are learning a novel task, lack accuracy and consistency in their performance, and therefore may hit the ball either too strongly or too weakly. If they hit it too strongly, the ball can go beyond the hole by a few (or more) centimeters, and if they hit it too weakly, the ball can stop a few (or more) centimeters in front of the hole. In these cases, it might be more appropriate to place the large circle as a visual aid either in back of or in front of the hole, depending on the golfer’s tendency to deviate from the target. Specifically, since the diameter of a golf ball is 4.27 cm and the golf hole diameter is 10.8 cm, providing a larger target (e.g., 20, 30, or 40 cm in diameter) may allow the golfer to direct the putt more easily to the desired location.

In previous research, a circular visual aid was placed only symmetrically around the golf hole, and therefore an examination of the effectiveness of various placements of the large circle around the target (in front of or behind the hole) in learning motor skills was not possible. Placing the circle in a certain location can bias participants to hit either somewhat strongly (if the circle is placed in back of the hole) or somewhat weakly (if the circle is placed in front of the hole). Indeed, placing a circular target in the back of the hole may improve putting accuracy. For example, a ball that is hit somewhat hard but accurately might still be holed. In addition, a specific directional bias can lead to improved putting consistency, as it may lead to putts being frequently directed to the same location. Importantly, putting consistency is an essential measure of motor learning, as suggested by Schmidt et al. (2018): “…the measure of error that is most sensitive to the effects of practice is consistency” (p. 28).

In addition, not only can the placement of the visual aid affect performance (e.g., a training effect observed in the acquisition phase; Soderstrom and Bjork, 2015) and learning (e.g., a relatively permanent change observed in retention and transfer tests; Soderstrom and Bjork, 2015) differently, but providing individuals with the autonomy to choose the placement of a visual aid may also affect performance and learning. This autonomy to choose for oneself has been proposed as an important contributor to skill acquisition (Wulf and Lewthwaite, 2016). Indeed, autonomy is one of three major concepts that make up the OPTIMAL (Optimizing Performance Through Intrinsic Motivation and Attention for Learning) theory of motor learning; the other two are *enhanced expectancies of success* and *external focus of attention* (Wulf and Lewthwaite, 2016). This theory suggests that applying any one of these concepts may enhance motor learning, and that their effects on motor learning can also be additive (Wulf et al., 2018).

A number of studies have already shown the benefits of providing autonomy to learners. In one study on bowling (Hooyman et al., 2014), autonomy-supportive instructions led to higher self-efficacy, positive affect, and improved retention performance, as compared to instructions given in a more controlling language. Providing autonomy appears to positively affect motor learning, even when it does not directly relate to the task at hand. For example, in one study (Lewthwaite et al., 2015, Exp. 1), participants in one group were given a choice of the color of the golf balls, while participants in the other group were linked to the first group and were provided with balls of the same color but without the ability to choose that color. The findings of this study showed that putting accuracy was significantly greater in the group that was provided with the autonomy to choose colors.

While exercising autonomy has been shown to be important in motor learning, individuals’ intuitive choices of accessories, aids, or cues may often hinder their performance in other domains of learning – a phenomenon called *preference-performance dissociation* (Andre and Wickens, 1995). This preference-performance dissociation has been observed in a number of domains associated with human factors, specifically in human-machine interface design. For example, Bailey (1993, Study 1) showed that a preference for interfaces in making grocery selections was dissociated from the time needed to complete the selections on those interfaces. A similar dissociation was reported when participants were asked about their preference for different types of widgets to control computer applications (Bailey, 1993, Study 2). Interestingly, in both studies the participants were computer professionals who were expected to have preferences that matched their performance.

In another study, Frøkjær et al. (2000) found weak correlations between effectiveness (i.e., accuracy and completeness of a task), efficiency (i.e., resources required to complete a task), and satisfaction (i.e., users’ positive attitudes toward the use of a system) in 87 undergraduate students who completed computerized information retrieval tasks.

The dissociation between preference and performance can be found in other domains as well. For example, in one study (Hegarty et al., 2012, Exp. 1) undergraduate students were asked to read weather maps. They were shown maps of various complexities, ranging from simple maps that included only the required information (e.g., wind direction, surface temperature) to the most complex maps that also included task-irrelevant information (e.g., terrain, state boundaries). While most participants chose to read the simplest map (67.3%), about 33% of the time participants chose maps with irrelevant geographic information. These extraneous map variables tended to slow down response time and increase the number of map-reading errors. In addition, gaze recording showed that with the more complex maps, more fixations were directed to both task-relevant and task-irrelevant areas of the map (Hegarty et al., 2012, Exp. 2). In another experiment on reading weather maps, post-graduate meteorology students also preferred maps with extraneous information about a third of the time, and due to this preference their performance suffered (Hegarty et al., 2012, Exp. 3).

In a randomized controlled trial from the domain of medicine (Ko et al., 2015), physicians were instructed to perform several motor tasks with a two-dimensional (2D) and a three-dimensional (3D) laparoscopic trainer. The skills included fundamental laparoscopic technical skills. The participants performed three tasks in a 2D laparoscope and the same three tasks in a 3D laparoscope in a counterbalanced manner. In general, the physicians took less time to perform the tasks and had higher self-evaluation scores when performing 3D laparoscopy. However, out of the 29 participating physicians, 11 (37.9%) preferred 2D laparoscopy. This percentage is similar to that found in the above-mentioned three-experiment study on weather-map reading.

In a study from another domain (Keyson and Parsons, 1990), professional ergonomists were asked to rate different computer menu interfaces. Three objective measures for each interface were used (i.e., time, number of keys, and errors). In addition, a one-on-one interview (participant-interface designer) was conducted. Five interfaces were examined by four ergonomists each, for a total of 20 participants. The results emerging from this study showed that the interface that led to the best performance (e.g., quickest reaction time, lowest number of errors) scored the lowest on subjective preference. In addition, the interface that scored *lowest* in performance was frequently the one that was preferred. As Andre and Wickens (1995) postulated, one possible explanation for the preference-performance dissociation is that people are unaware of the intricacies of human information processing mechanisms related to speed and accuracy in human-machine interaction. Finally, Wright and O’Hare (2015) found that, compared to an analog cockpit (i.e., a cockpit with dial instruments), glass cockpits (i.e., cockpits with large and colorful digital displays) were associated with poorer flight performance in novices, despite a strong subjective preference for glass cockpits. It is possible that it was not the type of instruments *per se* that affected performance, but it may have been the amount of information delivered in the glass cockpit that was too large for the participants to use effectively. However, regardless of the underlying reason for the participants’ preferences, there was a clear dissociation from performance.

It may be possible that such preference-performance dissociations occur when individuals are asked to assess what type of visual aid can help them perform and learn various closed, self-paced motor tasks. It is our assumption that preference-performance dissociations may also negatively influence motor skill acquisition in novice learners who aim at acquiring closed, self-paced sport skills, such as in a learning environment where different placements of visual aids (e.g., large circles around the target) are used.

In most studies on preference-performance dissociation, performance (rather than learning) was measured. As indicated previously, by *learning* we mean a relatively permanent change observed in advanced phases of practicing the putt. In our study, the advance phases were the retention and transfer. It has been well established in the literature on motor skill acquisition that learners can attain achievements differently in the performance phase (first phase of practice) than in later phases of practice (i.e., retention and transfer) (Soderstrom and Bjork, 2015). For example, learners can achieve better results in the retention and/or transfer phases than in the performance phase, because they gained some experience with the learned task in the early performance phase. Therefore, the constructs of *performance* and *learning* are dissimilar, and it is usually learning that is of most interest to coaches, instructors, and teachers (for a review on learning versus performance, see Soderstrom and Bjork, 2015). In addition, to the best of our knowledge there are no studies in motor learning that have directly examined whether participants will choose wisely when they are given the autonomy to do so.

Therefore, the purpose of this study was twofold: (a) to examine whether the phenomenon of preference-performance dissociation occurs when visual aids are placed in different locations around a target in the performance (i.e., acquisition phase) and learning (i.e., retention and transfer tests) of a task that requires accuracy (i.e., a golf putting task), and (b) to examine whether giving participants the choice of visual aid placement affects performance and learning. We hypothesized that there will be instances in which the participants’ subjective assessment of visual-aid helpfulness will contradict their actual performance and learning results.

## Materials and Methods

### Participants

Seventy-six physical education students (55 females and 21 males) with no experience in golf participated in the study (mean age = 23.3 ± 2.97 years). The participants were randomly assigned to four experimental groups: (a) a visual aid placed behind the hole (V-behind), (b) a visual aid placed in front of the hole (V-in-front), (c) a visual aid placed around the hole (V-around), and (d) a visual aid placed according to the participant’s preference (V-pref). Each group was composed of 19 participants. Randomization was conducted using a dedicated script written in Python. The participants were naïve to the purposes of the study and its assumptions. The Ethics Committee of The Academic College at Wingate approved the study.

### The Putting Task

**FIGURE 1:**
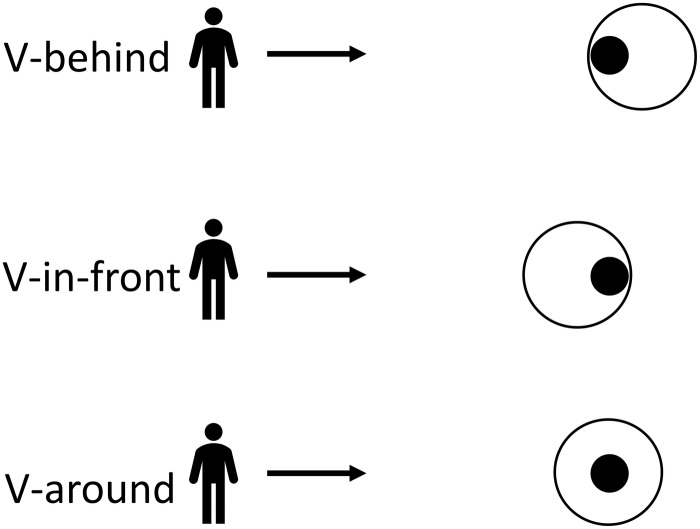
The three circle-placement options (fine line circles) around the golf hole (black-filled circle) for the V-behind, V-in-front, and V-around participants. (Participants in the V-pref group chose one of the three options).

The participants performed a putting task in three phases – acquisition, retention, and transfer. In the acquisition phase, they were asked to putt a regulation golf ball (diameter = 42.67 mm, mass = 45.93 g) toward a regulation golf hole (diameter = 10.8 cm) from a distance of 2 m on an indoor artificial putting green (1.22 × 4.27 m) (Birdie Ball, Inc., Evergreen, CO, United States). For the participants in the V-behind group, a 40-cm circle was placed tangent to the hole and away from the participant; for the participants in the V-around group, a 40-cm circle was placed evenly around the hole; and, for the participants in the V-in-front group, a 40-cm circle was placed tangent to the hole and toward the participant. The three circle placement options around the golf hole are presented in Figure 1. The participants in the V-pref group individually chose one of the three circle positions.

The circle was made of white cardboard and was attached to the green’s surface in such a way that it did not interfere with the ball’s trajectory. The task performed in the retention phase was similar to the one performed in the acquisition phase, however without the presence of the 40-cm circle around the hole. In the transfer phase, the putting distance was increased to 2.5 m and did not include the 40-cm circle.

### Procedure

Upon arrival at the Motor Behavior Laboratory, all participants signed an informed consent form. After the form was signed, the researcher taught the participants the techniques of golf putting. This teaching procedure was adopted since none of the participants played golf prior to the experiment, and they were completely naïve to the task. The instructions included the correct stance for putting a golf ball, with an emphasis on bent knees and a straight back, and the correct position and motion of the arms (see Pelz, 2005, 2012). Each participant then completed a pre-test of five trials without surrounding circles. Then, according to their group assignment, the participants performed five blocks of 10 putting trials. For the V-pref group, the choice of circle placement was recorded for each participant. Participants in all groups were instructed to try to hole the putt, and they were told that if the ball ended up resting within the 40-cm circle, the trial was considered to be successful. This was done in order to enhance the participants’ expectancies of success by providing an easier criterion for success. This strategy is often used in studies examining enhanced expectancies (e.g., Palmer et al., 2016; Ziv et al., 2019). After each putt, the researcher measured the X and Y coordinates of the ball location in relation to the center of the golf hole. The participants could see where the ball landed, but were not given any additional feedback regarding the distance (in centimeters) of the ball from the hole.

After completing the putts in the acquisition stage, the participants were asked whether they thought the visual aid had helped their performance during the five blocks of 10 putts [on a scale of 1 (not helpful) to 5 (very helpful)], and this information was recorded by the researcher. Forty-eight hours later, the participants performed the retention putts and the transfer putts. Each of the retention and transfer phases was composed of one block of 12 trials with no surrounding circles.

### Dependent Variables

Six dependent variables were measured: (a) Absolute Error (AE) – the deviation from the center of the hole in cm; (b) Bivariate Variable Error (VE) – a measure of putting consistency calculated as: $\text{VE}={\left\{\left(\frac{1}{k}\right)\,\sum_{i=1}^{k}\left[{\left(x_{i}-x_{c}\right)}^{2}+{\left(y_{i}-y_{c}\right)}^{2}\right]\right\}}^{1/2}$ (Hancock et al., 1995); (c) Absolute Constant Error for the *y*-axis (|CE|) – a measure of the distance from the center of the hole in cm, in which the sign is retained during calculation of the mean and the absolute value is added only after that calculation; (d) the number of putts landing within the circle (a discrete measure; only in the acquisition phase); (e) the number of putts landing in the golf hole (a discrete measure); and (f) a subjective assessment of circle-location helpfulness on a scale of 1 (not helpful for improving performance) to 5 (very helpful for improving performance).

In order to calculate the AE, VE, and |CE|, the *x* and *y* coordinates of each putt were measured in centimeters. For this purpose, the golf hole was considered to be the center of the *x* and *y* axes. If a ball came in contact with the rear border of the putting green or rolled passed the border of the putting green, a maximum measurable deviation of 60 cm was recorded.

### Statistical Analyses

AE, VE, |CE|, and the number of putts landing in the golf hole were calculated for the five pre-test trials, across the 10 trials for each block in the acquisition phase, and across the 12 trials of the retention and the 12 trials of the transfer phases. The number of putts landing within the circle was calculated only for the acquisition phase. A one-way analysis of variance (ANOVA) was performed to examine the pre-test data. A two-way ANCOVA (Group × Trial Block) with repeated measures on the Trial Block factor and the relevant pre-test variable as a covariate was performed to examine the data of the acquisition phase. Retention and transfer data were analyzed with an analysis of covariance (ANCOVA), where the pre-test performance was included as a covariate. A Bonferroni *post hoc* analysis was used when required.

A *t*-test was performed to analyze the performances of the participants in the V-in-front group, and of those in the V-pref group who chose to place the circle in front of the hole. We decided upon this statistical procedure post-experiment since 11 of the 19 participants in the V-pref group chose to place the circle in front of the hole, therefore allowing a decent sample size for such a comparison. The statistical level of significance for all analyses was set at *p* < 0.05, effect sizes were calculated as partial eta-squared (η^2^*_*p*_*), and all analyses were conducted using IBM SPSS Statistics version 25 (IBM Inc., United States). Lastly, normality of data was assessed by calculating skewness and kurtosis values. Data were considered to be normally distributed if both the skewness and kurtosis were between −2 and +2 (see Vincent, 2005).

## Results

**FIGURE 2:**
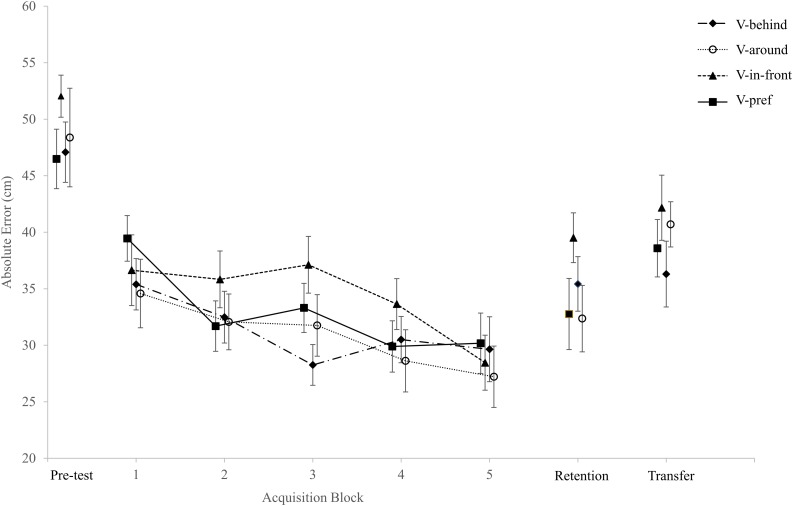
AE for the four experimental groups across the three phases of the study (means ± SE).

**FIGURE 3:**
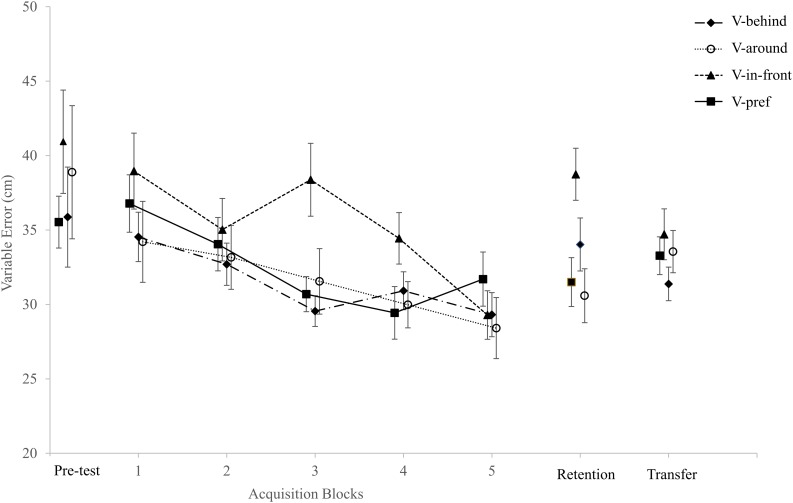
VE for the four experimental groups across the three phases of the study (means ± SE).

AE and VE for the four experimental groups across the three phases of the study are presented in Figures 2, 3, respectively. The dependent variables were normally distributed except for the VE in the pre-test, the VE in Block 3 of the acquisition stage, and the number of holed putts in Block 1 of acquisition. Since there were very few violations of normality we decided to use parametric statistics for all our analyses, despite the fact that some of our data were discrete (i.e., the number of balls landing within the circle and the number of holed putts).

### V-pref Group Preference for Circle Location

Out of the 19 participants in the V-pref group, three chose to place the visual aid behind the hole (16%), five chose to place it around the hole (26%), and 11 (58%) chose to place it in front of the hole.

### Subjective Assessment of the Helpfulness of Circle Location

A one-way ANOVA revealed a significant group difference, *F*(3,72) = 5.00, *p* = 0.003, η^2^*_*p*_* = 0.17. A Bonferroni *post hoc* analysis revealed that the subjective rating of the helpfulness of the circle location to improve performance was lower in the V-behind group (2.53 ± 1.43) compared to the V-in-front group (3.63 ± 0.96, *p* = 0.039) and the V-around group (3.63 ± 1.25, *p* = 0.039), but was not significantly different from the V-pref group (2.58 ± 1.17, *p* = 1.00). There was also a trend for the V-pref group rating to be lower than both the V-around and the V-in-front groups (in both: *p* = 0.056). There were no significant correlations between the subjective helpfulness score and the average AE, |CE|, VE, the number of balls landing within the circle, or the number of holed putts (*r* < |0.21|).

### Pre-test

The one-way ANOVA revealed no significant differences between experimental groups for AE, *F*(3,72) = 0.68, *p* = 0.57, VE, *F*(3,72) = 0.57, *p* = 0.64, |CE|, *F*(3,72) = 0.31, *p* = 0.82, and the number of holed putts, *F*(3,72) = 1.94, *p* = 0.13.

### Acquisition Phase

#### AE

The two-way ANCOVA (Group × Trial Block) with repeated measures on the Trial Block factor and the pre-test AE as the covariate revealed a main effect for Trial Block, *F*(4,284) = 2.63, *p* = 0.035, η^2^*_*p*_* = 0.04. A Bonferroni *post hoc* analysis revealed that the AE of Block 5 (28.87 ± 11.47 cm) was lower than the AE of Block 1 (36.52 ± 11.50 cm), Block 2 (33.02 + 10.29 cm) and Block 3 (32.61 ± 10.44 cm), but not significantly different than that of Block 4 (30.66 ± 10.18 cm). The AE of Block 4 was lower than that of Block 1 only. There was no significant main effect for Group, *F*(3,71) = 0.57, *p* = 0.64, η^2^*_*p*_* = 0.02, and no significant interaction, *F*(12,284) = 1.28, *p* = 0.23, η^2^*_*p*_* = 0.05.

#### VE

After correcting for sphericity, the two-way ANCOVA (Group × Trial Block) with repeated measures on the Trial Block factor, and the pre-test VE as the covariate, revealed no significant differences for Trial Block, *F*(3.52,250.13) = 1.33, *p* = 0.26, η^2^_*p*_ = 0.02, no significant main effect for Group, *F*(3,71) = 1.97, *p* = 0.13 η^2^*_*p*_* = 0.08, and no significant interaction, *F*(10.60,250.13) = 1.1, *p* = 0.36, η^2^*_*p*_* = 0.04.

#### |CE|

The two-way ANCOVA (Group × Trial Block) with repeated measures on the Trial Block factor and the pre-test |CE| as the covariate revealed a main effect for Trial Block, *F*(4,284) = 2.9, *p* = 0.02, η^2^*_*p*_* = 0.04. However, a Bonferroni *post hoc* analysis failed to reveal any differences between blocks. Neither the main effect for Group, *F*(3,71) = 1.04, *p* = 0.38, η^2^*_*p*_* = 0.04, nor the interaction (Group × Trial Block), *F*(12,284) = 1.02, *p* = 0.43, η^2^*_*p*_* = 0.04, was found to be significant.

#### Number of Balls Landing Within the Circle

The two-way ANCOVA (Group × Trial Block) with repeated measures on the Trial Block factor and the pre-test number of balls landing within the circle as a covariate revealed a main effect for Trial Block, *F*(4,284) = 4.58, *p* = 0.001, η^2^*_*p*_* = 0.06. A Bonferroni *post hoc* analysis revealed that the number of balls landing within the 40-cm circle was smaller in Block 1 (2.84 ± 2.05) compared to that of Block 2 (3.64 ± 1.96), Block 4 (3.89 ± 1.89), and Block 5 (4.07 ± 2.33), but not compared to that of Block 3 (3.34 ± 1.87). The Group main effect, *F*(3,71) = 0.72, *p* = 0.55, η^2^*_*p*_* = 0.03, and the interaction, *F*(12,284) = 1.02, *p* = 0.39, η^2^*_*p*_* = 0.04, were not found to be significant.

#### Number of Holed Putts

After correcting for sphericity, the two-way ANCOVA (Group × Trial Block) with repeated measures on the Trial Block factor and the pre-test holed putts as a covariate revealed no statistically significant differences for Trial Block, *F*(3.57,253.84) = 1.64, *p* = 0.17, η^2^*_*p*_* = 0.02, no significant main effect for Group, *F*(3,71) = 0.74, *p* = 0.53, η^2^*_*p*_* = 0.03, and no significant interaction, *F*(10.72,253.84) = 0.94, *p* = 0.51, η^2^*_*p*_* = 0.04.

### Retention Phase

One-way ANCOVAs with pre-test AE, VE, |CE|, and balls landing in the hole variables as covariates were performed on the retention AE, VE, |CE|, and balls landing in the hole variables, respectively. The analysis failed to find significant differences between groups in AE, *F*(3,71) = 1.15, *p* = 0.33, η^2^*_*p*_* = 0.05, |CE|, *F*(3,71) = 0.4, *p* = 0.75, η^2^*_*p*_* = 0.02, or in the number of balls landing in the hole, *F*(3,71) = 1.41, *p* = 0.25, η^2^*_*p*_* = 0.06. However, a significant difference between groups was found for VE, *F*(3,71) = 4.11, *p* = 0.01, η^2^*_*p*_* = 0.15. A Bonferroni *post hoc* analysis revealed that the VE in the V-in-front group (38.74 ± 7.64 cm) was higher than the VE in the V-around group (30.56 ± 7.89 cm) and V-pref group (31.50 ± 8.11 cm), but not significantly different from the VE in the V-behind group (34.03 ± 7.77 cm). No significant differences between groups were found for the number of balls landing in the hole.

To further assess the differences in VE between groups, we took only those participants in the V-pref group who chose to place the ball in front of the hole (*n* = 11) and compared them with the participants in the V-in-front group (*n* = 19). An independent *t*-test indicated that the VE of the V-in-front group (38.74 ± 7.64 cm) was higher than the VE of the participants in the V-pref group who chose the same circle location (32.36 ± 6.85 cm), *t*(28) = 2.29, *p* = 0.03, Cohen’s *d* = 0.84.

### Transfer Phase

The one-way ANCOVA with the pre-test performance as the covariate failed to find significant differences between groups in AE, *F*(3,71) = 0.65, *p* = 0.59, η^2^*_*p*_* = 0.03, VE, *F*(3,71) = 1.14, *p* = 0.34, η^2^*_*p*_* = 0.05, |CE|, *F*(3,71) = 0.09, *p* = 0.97, η^2^*_*p*_* = 0.00, or in the number of balls landing in the hole, *F*(3,71) = 1.00, *p* = 0.39, η^2^*_*p*_* = 0.04.

### Pre-test Versus Block 1 of Acquisition

A two-way ANOVA (Group × Stage) with repeated measures on the stage factor (pre-test versus Block 1 of acquisition) was performed in order to assess the effects of adding a visual aid to the putting task.

For AE, a significant difference between the pre-test (48.49 ± 13.09 cm) and Block 1 of acquisition (36.52 ± 11.50 cm) was found, *F*(1,72) = 55.84, *p* < 0.001, η^2^*_*p*_* = 0.44. There were no significant differences between groups, *F*(3,72) = 0.38, *p* = 0.77, η^2^*_*p*_* = 0.02, and no significant interaction, *F*(3,72) = 1.28, *p* = 0.29, η^2^*_*p*_* = 0.05.

For VE, there were no significant group differences, *F*(3,72) = 0.95, *p* = 0.42, η^2^*_*p*_* = 0.04, no significant stage differences, *F*(1,72) = 0.74, *p* = 0.39, η^2^*_*p*_* = 0.01, and no significant interaction, *F*(3,72) = 0.39, *p* = 0.76, η^2^*_*p*_* = 0.02.

For |CE|, a significant difference between the pre-test (30.51 ± 15.79 cm) and Block 1 of acquisition (18.34 ± 11.61 cm) was found, *F*(1,72) = 33.09, *p* < 0.001, η^2^*_*p*_* = 0.32. There were no significant differences between groups, *F*(3,72) = 0.86, *p* = 0.47, η^2^*_*p*_* = 0.01 and no significant interaction, *F*(3,72) = 0.14, *p* = 0.94, η^2^*_*p*_* = 0.01.

## Discussion

The purpose of the current study was to examine the possible effects of providing autonomy and of the phenomenon of preference-performance dissociation on a closed, self-paced motor task – putting in golf. We hypothesized that (a) providing autonomy will lead to improved performance and learning, and that (b) there will be a dissociation between participants’ preference or subjective assessment of visual aids and their ability to perform.

The findings of the current study partially supported the first hypothesis. While putting accuracy (i.e., AE, |CE|) did not differ between the four experimental conditions throughout the study, putting consistency (i.e., VE) in the retention phase was inferior in the V-in-front group compared to the V-around and the V-pref groups. More importantly, when comparing putting consistency of the participants in the V-in-front group to that of the participants in the V-pref group who chose to place the circle in front of the golf hole, the difference was still significant. The latter finding suggests that providing participants with the autonomy to choose circle placement alleviated the reduced putting consistency seen in the V-in-front group.

The second hypothesis was also partially supported by our data. The fact that the participants in the V-in-front group assumed that the visual aid helped them to attain achievement, even though their putting consistency was the lowest among the four experimental groups in the retention test (and actually deteriorated from the last block of acquisition), is an example of the phenomenon of preference-performance dissociation.

We discuss first the possibility that the finding of putting consistency (VE) may have been a result of a type I error, and then discuss the difference in putting consistency in terms of providing autonomy and the preference-performance dissociation. For the potential threat of type I error, we propose four explanations. First, the moderate effect sizes in the ANCOVA (η^2^*_*p*_* = 0.15) and in the *t*-test (Cohen’s *d* = 0.84) suggest that this finding is practically meaningful. Second, we took the precautions of using ANCOVA with the pre-test VE as a covariate and performing a Bonferroni *post hoc* analysis for group comparisons. Third, we applied a multiple comparison method – the False Discovery Rate (Benjamini and Hochberg, 1995; Pike, 2011) – for the four-group comparison of VE (i.e., pre-test VE, acquisition group effect of VE, retention VE, and transfer VE). The observed difference in the retention VE between groups remained significant. Fourth, VE is a sensitive measure in motor skill acquisition (Schmidt et al., 2018), and therefore this measure is likely to differentiate between experimental groups sooner than other measures. These four explanations suggest that this finding is likely to be valid.

Our findings also showed improvement in AE and |CE| (but not in VE) in all groups between the pre-test and Block 1 of acquisition. These findings suggest that adding a visual aid (which was present in Block 1 of acquisition but not in the pre-test) may lead to improved accuracy. However, as VE did not change, it is possible that adding a visual aid is not enough to improve putting consistency in early stages of practice. It is also very likely that the differences between the pre-test and Block 1 of acquisition are simply a part of the learning process, and would have been found regardless the presence of the visual aid. This explanation should be examined in future studies.

The contribution of autonomy to motor skill acquisition has been examined in previous studies, and has been incorporated into one theory of motor learning – the OPTIMAL theory (Wulf and Lewthwaite, 2016). Indeed, the ability to have control over one’s own environment is a basic psychological need (Deci and Ryan, 2008), and appears to be a biologically motivated necessity (Leotti et al., 2010; Leotti and Delgado, 2011). In this respect, a number of studies have shown that various types of autonomy can enhance motor learning and performance. For example, when participants control the timing of receiving feedback about their performance, they tend to perform better than when the feedback schedule is decided upon by someone else. This is true for the two general types of feedback: knowledge of performance (Janelle et al., 1997) and knowledge of results (Patterson and Carter, 2010). In addition, it was found that providing participants with the autonomy to choose when to use a physical assistive device when trying to maintain balance also appears to facilitate learning (Hartman, 2007).

Even when the provided autonomy is not directly related to the task, performance and learning appear to improve. For example, golf-putting performance in a 24-h delayed retention test was better in a group of participants who chose the color of the golf ball during training than in a group of yoked participants (Lewthwaite et al., 2015, Exp. 1). In addition, compared to a group of yoked participants, better performance in a retention test of a balance task was also reported in a group of participants who were asked which task they wanted to perform after the balance task, as well as which Renoir prints the researcher should hang on the laboratory wall (Lewthwaite et al., 2015, Exp. 2). The findings of the current study add to the literature on autonomy by showing that giving participants the opportunity to choose the location of the visual aid alleviates the reduced putting consistency associated with using the same location as dictated by the researcher.

One of the mechanisms that can explain the effects of providing autonomy, that is, providing participants with choice, on motor performance is increased self-efficacy. Indeed, having autonomy or control over one’s environment may increase participants’ self-efficacy (Wulf et al., 2014). Such higher self-efficacy, in turn, can be a predictor of success in future retention and transfer performances (Feltz et al., 2008; Pascua et al., 2015). Pascua et al. (2015) also suggested that increased self-efficacy can be a self-fulfilling prophecy. In essence, confidence in one’s ability to perform well enables the person to actually do so. This notion is also supported by the concept of psychological suggestion (Michael et al., 2012), which proposes that what people believe, think, or feel can influence the way they behave. Unfortunately, self-efficacy was not measured in the current study. Therefore, we cannot ascertain whether this was one of the underlying mechanisms responsible for the findings obtained in our study. It is suggested that in future studies, researchers assess the participants’ subjective self-efficacy.

While providing autonomy appears to lead to improved performance in motor learning, to the best of our knowledge there are no studies that directly examined whether participants will choose wisely when they are given the autonomy to do so. More specifically, in the context of the current study, it is not known whether the participants’ choice for the location of a visual aid will indeed facilitate better performance. Our findings suggest that while providing autonomy is useful for motor learning, under certain conditions the participants’ subjective assessment of visual aid helpfulness may be erroneous. The participants in the V-in-front and the V-around groups perceived the visual aid as more helpful than the participants in the V-behind and V-pref groups. However, putting consistency of the participants in the V-in-front was the lowest among the four experimental groups in the retention test. This dissociation was true for the V-pref group as well. For the participants in the V-pref group, while the subjective rating of the helpfulness of the visual aid was low, putting consistency in the retention test was as good as that of the participants in the V-around and V-behind groups. Indeed, eight out of 11 (73%) participants from the V-pref group who chose to place the visual aid in front of the hole rated its helpfulness as three or below (out of five). However, it should be noted that the lowest values of the subjective assessment of the helpfulness of circle locations were above 2.50, so the margin between these values and the highest value (above 3.60) was relatively small. Therefore, we should adopt a careful approach when interpreting these subjective data.

The preference-performance dissociation phenomenon has been shown in various studies in the field of usability (e.g., Hegarty et al., 2012; Roberts et al., 2017). For example, Roberts et al. (2017, Exp. 1) showed that a curvilinear metro map allowed for better trip planning performance when compared to the more common octolinear map. However, the participants’ map preference was not related to performance. While 63 out of 80 participants performed better with the curvilinear map, the subjective rating of map preference was almost equal between the two types of maps. Since this was a repeated-measures design, the authors were able to conclude that performing journey planning with both maps did not affect map preference. The fact that objective usability (e.g., planning duration) was unrelated to preference was not surprising, since being aware of a difference of only a few seconds between designs may have been too subtle for the participants to notice (Roberts et al., 2017). Hegarty et al. (2012) reported an even more distinct preference-performance dissociation when reading weather maps. In their study, the subjective choice of maps tended to lead to reduced performance.

However, in these usability studies the effects of different perceptual cues on cognitive performance were also examined (e.g., route planning, map reading). In contrast, in motor learning in general and in golf putting in particular, it is the perceptual-motor system that is being challenged. In other words, it is the cycle of perception and action that is of interest when learning a motor task; that is, we are looking at the effects of the visual aid location (perception) on putting performance (action). Only a small number of studies have examined the preference-performance dissociation in this context (e.g., Ko et al., 2015; Wright and O’Hare, 2015), and none of them used sport-related tasks. In addition, most of those studies did not examine autonomy. The literature on autonomy, which is also available in sport-related tasks (e.g., Hooyman et al., 2014; Lewthwaite et al., 2015), appears to be distinct from the preference-performance dissociation literature.

Our study attempted to examine the possible contradictions between the provision of autonomy and the preference-performance dissociation. However, only VE during retention was affected by both. This can be explained by the fact that VE is the measure most sensitive to practice (Schmidt et al., 2018). Our participants practiced a novel task in only 50 trials. It is possible that differences in AE and holed putts would have become apparent only after additional practice. Additional studies are needed to directly assess the effects of different preferences on performance.

The preferences of the V-pref group were somewhat surprising. We intuitively expected that most of the participants would choose to place the circle symmetrically around the hole. However, only five participants in the V-pref group (26%) chose this location, while 11 chose to place it in front of the hole. This choice led, at the same time, to better consistency compared to the V-in-front group, as well as to reduced subjective assessment of the helpfulness of this visual aid.

As far as we know, the current study is the first to show that a certain type of preference-performance dissociation may occur in motor learning – specifically in learning closed, self-paced motor skills. While allowing participants to choose the location of the circle led to better performance, their subjective assessment of the circle location did not necessarily match their putting consistency. The possibility that this phenomenon exists suggests that while providing autonomy to learners can be of great benefit, caution should be taken to ensure that participants choose what is actually good for them.

It should be noted that in a “classic” *preference-performance dissociation*, the preference is dissociated from performance rather than from learning. However, in the current study, the subjective assessment of the helpfulness of the visual aid was obtained from the participants directly after the acquisition phase, while the dissociation from performance was observed only during the retention test.

There are two possible explanations for this finding. First, while the subjective rating of visual aid helpfulness was higher in the V-in-front group and the V-around group compared to the V-behind group, it did not result in better performance. While this is not a robust finding (i.e., the higher assessment of helpfulness was not accompanied by reduced performance), it may still suggest that individuals do not always value what helps them to attain achievement. Second, there are instances that the preference during practice manifests itself only in the retention test. For example, one survey (McCabe, 2011) showed that over 90% of students preferred massed studying over distributed studying when a learning scenario was presented to them. This preference would only manifest itself in a later retention test (e.g., an actual classroom test) and not during practice. Indeed, it has been previously shown that distributed practice supports learning better than massed practice. For example, a recent systematic review of surgical skills has shown that, compared to massed practice, distributed (or spaced) practice promotes long-term skill retention (Cecilio-Fernandes et al., 2018).

It can then be argued that the *preference-performance dissociation* can be expanded and that perhaps a *preference-learning dissociation* exists. This should be examined in additional studies on a number of known learning strategies, among them contextual interference, and on various feedback strategies in which participants’ preferences during task acquisition may be dissociated from the benefits for long-term learning.

Three limitations of the current study are noteworthy. First, the between-subject design did not allow all participants to practice all of the visual aid locations. A within-subject design that included a preference questionnaire may have allowed for a better understanding of the preference-performance dissociation. This could have been complemented with a pre-test of performance composed of the three visual-aid locations. However, such a design would not have allowed us to examine the differences in the learning process. That is to say, if all participants practiced all the conditions, the retention and transfer tests would not have had any meaning. Therefore, as this was a learning rather than a performance study, such a design was unwarranted. Still, future studies should attempt to assess performance directly by using a within-subject design, as well as by comparing visual-aid preference to pre-test performance with those same visual aids.

Second, only the participants in the V-pref group were asked where they wanted to place the visual aid. However, the sample size of this group (*n* = 19) was not large enough to directly compare preferences and performance. Hence, our measure for preference (i.e., helpfulness) was an indirect one. We propose that in a future between-subject design study, all of the participants will be asked for their preference. In such a study, participants would be recruited until the sample size of all the groups is large enough to provide ample statistical power.

Third, the participants in our study completed a pre-test of five trials without surrounding circles. This number of baseline trials might not be enough. In the future, it may be prudent to provide participants with additional baseline trials in order to ensure that indeed the autonomy would have led to improved performance and learning.

## Conclusion

The findings of this study suggest that golf-putting consistency measured in a 48-h delayed retention test can benefit from allowing participants to choose the placement of a visual aid around the golf hole. However, while the measure of helpfulness was indirect – and therefore this conclusion should be considered with caution, individuals’ subjective assessment of the helpfulness of this visual aid may contradict actual putting consistency. The possible dissonance between providing autonomy and the preference-performance dissociation should be made known to coaches and instructors. Indeed, instructors and coaches in golf should be aware that while the provision of autonomy to players can facilitate motor learning, players who are provided with this autonomy might not always choose correctly. In future studies on preference-performance dissociation and autonomy in learning closed, self-paced motor tasks (e.g., putting in golf), we propose that researchers also use a qualitative assessment (e.g., in-depth interviews, observations) that can reveal the reasons for choosing the placement of the visual aid. Such an assessment may provide insights into the participants’ thought processes when choosing the location of the visual aid. In addition, assessment of gaze behavior and kinematic variables, as well as interventions of longer durations, could increase our understanding of this instructional issue.

## Data Availability Statement

The datasets generated for this study are available on request to the corresponding author.

## Ethics Statement

This study was carried out in accordance with the recommendations of the Ethics Committee at The Academic College at Wingate with written informed consent from all subjects. All subjects gave written informed consent in accordance with the Declaration of Helsinki. The protocol was approved by the Ethics Committee at The Academic College at Wingate.

## Author Contributions

GZ and RL designed the study. GZ conducted the data analysis and wrote most of the manuscript. RL contributed to writing sections of the manuscript and editing. ML and LE conducted the experiment and commented on the manuscript.

## Conflict of Interest

The authors declare that the research was conducted in the absence of any commercial or financial relationships that could be construed as a potential conflict of interest.
